# Supplementary material for: Genetic Susceptibility of HLA Alleles to Non-Steroidal Anti-Inflammatory Drug Hypersensitivity in the Taiwanese Population
Source: Biomedicines. 2023 Dec 11;11(12):3273. doi: 10.3390/biomedicines11123273 (PMC10741656; doi:10.3390/biomedicines11123273)
Supplement: Supplementary file 1 [file biomedicines-11-03273-s001.zip › Supplementary Tables.pdf]

Supplementary Table S1. The genotype frequencies of the *HLA-A* alleles in the participants.

Variables		NSAID allergy n = 2434 (alleles)		Control n = 24340 (alleles)		*p* value[a]
		n	%	n	%	
A*01:01	No	2421	99.5	24176	99.3	
	Yes	13	0.5	164	0.7	0.497
A*02:01	No	2146	88.2	21958	90.2	
	Yes	288	11.8	2382	9.8	0.002
A*02:03	No	2301	94.5	22928	94.2	
	Yes	133	5.5	1412	5.8	0.526
A*02:05	No	2431	99.9	24334	100	
	Yes	3	0.1	6	0	0.051
A*02:06	No	2363	97.1	23616	97	
	Yes	71	2.9	724	3	0.923
A*02:07	No	2177	89.4	21722	89.2	
	Yes	257	10.6	2618	10.8	0.791
A*03:01	No	2426	99.7	24251	99.6	
	Yes	8	0.3	89	0.4	0.910
A*11:01	No	1709	70.2	17186	70.6	
	Yes	725	29.8	7154	29.4	0.701
A*11:02	No	2329	95.7	23014	94.6	
	Yes	105	4.3	1326	5.4	0.020
A*11:03	No	2434	100	24337	100	
	Yes	0	0	3	0	1.000
A*24:02	No	2036	83.6	20191	83	
	Yes	398	16.4	4149	17	0.400
A*24:10	No	2433	100	24333	100	
	Yes	1	0	7	0	1.000
A*24:353	No	2429	99.8	24300	99.8	
	Yes	5	0.2	40	0.2	0.832
A*26:01	No	2369	97.3	23642	97.1	
	Yes	65	2.7	698	2.9	0.622
A*29:01	No	2432	99.9	24303	99.8	
	Yes	2	0.1	37	0.2	0.560
A*30:01	No	2403	98.7	24014	98.7	
	Yes	31	1.3	326	1.3	0.860
A*31:01	No	2377	97.7	23833	97.9	

	Yes	57	2.3	507	2.1	0.439
A*32:01	No	2428	99.8	24270	99.7	
	Yes	6	0.2	70	0.3	0.870
A*33:01	No	2433	100	24339	100	
	Yes	1	0	1	0	0.434
A*33:03	No	2181	89.6	21768	89.4	
	Yes	253	10.4	2572	10.6	0.818
A*34:01	No	2430	99.8	24336	100	
	Yes	4	0.16	4	0.016	0.001
A*68:01	No	2433	100	24312	99.9	
	Yes	1	0	28	0.1	0.463
A*68:02	No	2433	100	24339	100	
	Yes	1	0	1	0	0.434
A*74:02	No	2429	99.8	24319	99.9	
	Yes	5	0.2	21	0.1	0.145
A*74:05	No	2433	100	24339	100	
	Yes	1	0	1	0	0.434

[a] Categorical variables were expressed as numbers (percent) and were analyzed using the Chi-square test.

Supplementary Table S2. The genotype frequencies of the *HLA-B* alleles in the participants.

Variables		NSAID allergy n = 2434 (alleles)		Control n = 24340 (alleles)		*p* value[a]
		n	%	n	%	
B*07:02	No	2427	99.7	24248	99.6	
	Yes	7	0.3	92	0.4	0.599
B*07:05	No	2431	99.9	24308	99.9	
	Yes	3	0.1	32	0.1	1.000
B*07:06	No	2433	100	24339	100	
	Yes	1	0	1	0	0.434
B*08:01	No	2423	99.5	24287	99.8	
	Yes	11	0.5	53	0.2	0.042
B*13:01	No	2265	93.1	22754	93.5	
	Yes	169	6.9	1586	6.5	0.442
B*13:02	No	2406	98.8	23976	98.5	
	Yes	28	1.2	364	1.5	0.207
B*14:02	No	2433	100	24339	100	
	Yes	1	0	1	0	0.434
B*15:01	No	2350	96.5	23368	96	
	Yes	84	3.5	972	4	0.209
B*15:02	No	2343	96.3	23327	95.8	
	Yes	91	3.7	1013	4.2	0.343
B*15:11	No	2419	99.4	24177	99.3	
	Yes	15	0.6	163	0.7	0.858
B*15:17	No	2434	100	24336	100	
	Yes	0	0	4	0	1.000
B*15:18	No	2413	99.1	24183	99.4	
	Yes	21	0.9	157	0.6	0.259
B*15:19	No	2431	99.9	24319	99.9	
	Yes	3	0.1	21	0.1	0.821
B*15:25	No	2410	99	24132	99.1	
	Yes	24	1	208	0.9	0.581
B*15:27	No	2428	99.8	24231	99.6	
	Yes	6	0.2	109	0.4	0.199
B*15:32	No	2433	100	24330	100	
	Yes	1	0	10	0	1.000
B*18:01	No	2427	99.7	24282	99.8	

	Yes	7	0.3	58	0.2	0.799
B*27:04	No	2336	96	23436	96.3	
	Yes	98	4	904	3.7	0.473
B*27:05	No	2432	99.9	24319	99.9	
	Yes	2	0.1	21	0.1	1.000
B*27:07	No	2434	100	24339	100	
	Yes	0	0	1	0	1.000
B*35:01	No	2361	97	23685	97.3	
	Yes	73	3	655	2.7	0.409
B*35:02	No	2432	99.9	24323	99.9	
	Yes	2	0.1	17	0.1	1.000
B*35:03	No	2431	99.9	24274	99.7	
	Yes	3	0.1	66	0.3	0.245
B*35:05	No	2434	100	24338	100	
	Yes	0	0	2	0	1.000
B*37:01	No	2423	99.5	24237	99.6	
	Yes	11	0.5	103	0.4	0.965
B*38:01	No	2434	100	24338	100	
	Yes	0	0	2	0	1.000
B*38:02	No	2328	95.6	23396	96.1	
	Yes	106	4.4	944	3.9	0.271
B*39:01	No	2368	97.3	23700	97.4	
	Yes	66	2.7	640	2.6	0.861
B*40:01	No	1905	78.3	19128	78.6	
	Yes	529	21.7	5212	21.4	0.733
B*40:02	No	2384	97.9	23756	97.6	
	Yes	50	2.1	584	2.4	0.318
B*40:03	No	2434	100	24339	100	
	Yes	0	0	1	0	1.000
B*40:06	No	2398	98.5	24040	98.8	
	Yes	36	1.5	300	1.2	0.344
B*42:01	No	2434	100	24335	100	
	Yes	0	0	5	0	1.000
B*44:02	No	2428	99.8	24286	99.8	
	Yes	6	0.2	54	0.2	0.984
B*44:03	No	2425	99.6	24243	99.6	
	Yes	9	0.4	97	0.4	0.963
B*46:01	No	2131	87.6	21120	86.8	

	Yes	303	12.4	3220	13.2	0.292
B*48:01	No	2385	98	23917	98.3	
	Yes	49	2	423	1.7	0.366
B*48:03	No	2434	100	24339	100	
	Yes	0	0	1	0	1.000
B*49:01	No	2434	100	24338	100	
	Yes	0	0	2	0	1.000
B*50:01	No	2431	99.9	24325	99.9	
	Yes	3	0.1	15	0.1	0.479
B*51:01	No	2335	95.9	23219	95.4	
	Yes	99	4.1	1121	4.6	0.245
B*51:02	No	2408	98.9	24075	98.9	
	Yes	26	1.1	265	1.1	1.000
B*51:07	No	2430	99.8	24302	99.8	
	Yes	4	0.2	38	0.2	1.000
B*52:01	No	2412	99.1	24124	99.1	
	Yes	22	0.9	216	0.9	1.000
B*54:01	No	2357	96.8	23494	96.5	
	Yes	77	3.2	846	3.5	0.455
B*55:01	No	2434	100	24337	100	
	Yes	0	0	3	0	1.000
B*55:02	No	2335	95.9	23486	96.5	
	Yes	99	4.1	854	3.5	0.173
B*55:04	No	2428	99.8	24318	99.9	
	Yes	6	0.2	22	0.1	0.052
B*55:07	No	2434	100	24338	100	
	Yes	0	0	2	0	1.000
B*56:01	No	2416	99.3	24229	99.5	
	Yes	18	0.7	111	0.5	0.076
B*56:03	No	2423	99.5	24259	99.7	
	Yes	11	0.5	81	0.3	0.438
B*56:04	No	2432	99.9	24308	99.9	
	Yes	2	0.1	32	0.1	0.724
B*57:01	No	2433	100	24288	99.8	
	Yes	1	0	52	0.2	0.113
B*58:01	No	2190	90	21838	89.7	
	Yes	244	10	2502	10.3	0.719
B*67:01	No	2427	99.7	24258	99.7	

	Yes	7	0.3	82	0.3	0.827

[a] Categorical variables were expressed as numbers (percent) and were analyzed using the Chi-square test.

Supplementary Table S3. The genotype frequencies of the *HLA-C* alleles in the participants.

**Variables**		**NSAID allergy n = 2434 (alleles)**		**Control n = 24340 (alleles)**		***p* value[a]**
		**n**	**%**	**n**	**%**	
C*01:02	No	1953	80.2	19515	80.2	
	Yes	481	19.8	4825	19.8	0.963
C*02:02	No	2431	99.9	24314	99.9	
	Yes	3	0.1	26	0.1	1.000
C*03:02	No	2192	90.1	21835	89.7	
	Yes	242	9.9	2505	10.3	0.613
C*03:03	No	2323	95.4	23194	95.3	
	Yes	111	4.6	1146	4.7	0.781
C*03:04	No	2110	86.7	21220	87.2	
	Yes	324	13.3	3120	12.8	0.509
C*04:01	No	2348	96.5	23383	96.1	
	Yes	86	3.5	957	3.9	0.361
C*04:03	No	2391	98.2	23922	98.3	
	Yes	43	1.8	418	1.7	0.923
C*04:82	No	2418	99.3	24190	99.4	
	Yes	16	0.7	150	0.6	0.912
C*05:01	No	2427	99.7	24287	99.8	
	Yes	7	0.3	53	0.2	0.638
C*06:02	No	2391	98.2	23805	97.8	
	Yes	43	1.8	535	2.2	0.186
C*07:01	No	2434	100	24329	100	
	Yes	0	0	11	0	0.600
C*07:02	No	1898	78	19142	78.6	
	Yes	536	22	5198	21.4	0.461
C*07:04	No	2410	99	24184	99.4	
	Yes	24	1	156	0.6	0.063
C*07:06	No	2428	99.8	24294	99.8	
	Yes	6	0.2	46	0.2	0.709
C*07:66	No	2434	100	24339	100	
	Yes	0	0	1	0	1.000
C*08:01	No	2245	92.2	22525	92.5	
	Yes	189	7.8	1815	7.5	0.610
C*08:02	No	2433	100	24339	100	

	Yes	1	0	1	0	0.434
C*08:03	No	2433	100	24319	99.9	
	Yes	1	0	21	0.1	0.711
C*08:22	No	2428	99.8	24254	99.6	
	Yes	6	0.2	86	0.4	0.498
C*12:02	No	2314	95.1	23211	95.4	
	Yes	120	4.9	1129	4.6	0.548
C*12:03	No	2418	99.3	24127	99.1	
	Yes	16	0.7	213	0.9	0.319
C*14:02	No	2362	97	23446	96.3	
	Yes	72	3	894	3.7	0.081
C*14:03	No	2433	100	24317	99.9	
	Yes	1	0	23	0.1	0.628
C*15:02	No	2333	95.9	23357	96	
	Yes	101	4.1	983	4	0.833
C*15:05	No	2434	100	24339	100	
	Yes	0	0	1	0	1.000
C*16:02	No	2429	99.8	24315	99.9	
	Yes	5	0.2	25	0.1	0.260
C*17:01	No	2434	100	24338	100	
	Yes	0	0	2	0	1.000

[a] Categorical variables were expressed as numbers (percent) and were analyzed using the Chi-square test.

Supplementary Table S4. The genotype frequencies of the *HLA-DPA1* alleles in the participants.

Variables		NSAID allergy n = 2434 (alleles)		Control n = 24340 (alleles)		*p* value[a]
		n	%	n	%	
DPA1*01:03	No	1814	74.5	17698	72.7	
	Yes	620	25.5	6642	27.3	0.058
DPA1*02:01	No	2234	91.8	22448	92.2	
	Yes	200	8.2	1892	7.8	0.461
DPA1*02:02	No	931	38.2	9691	39.8	
	Yes	1503	61.8	14649	60.2	0.138
DPA1*02:07	No	2389	98.2	23826	97.9	
	Yes	45	1.8	514	2.1	0.429
DPA1*04:01	No	2368	97.3	23697	97.4	
	Yes	66	2.7	643	2.6	0.890

[a] Categorical variables were expressed as numbers (percent) and were analyzed using the Chi-square test.

Supplementary Table S5. The genotype frequencies of the *HLA-DPB1* alleles in the participants.

Variables		NSAID allergy n = 2434 (alleles)		Control n = 24340 (alleles)		*p* value[a]
		n	%	n	%	
DPB1*01:01	No	2430	99.8	24313	99.9	
	Yes	4	0.2	27	0.1	0.670
DPB1*02:01	No	2087	85.7	20661	84.9	
	Yes	347	14.3	3679	15.1	0.271
DPB1*02:02	No	2272	93.3	22599	92.8	
	Yes	162	6.7	1741	7.2	0.385
DPB1*03:01	No	2322	95.4	23165	95.2	
	Yes	112	4.6	1175	4.8	0.655
DPB1*04:01	No	2279	93.6	22523	92.5	
	Yes	155	6.4	1817	7.5	0.053
DPB1*04:02	No	2378	97.7	23885	98.1	
	Yes	56	2.3	455	1.9	0.160
DPB1*05:01	No	1279	52.5	13216	54.3	
	Yes	1155	47.5	11124	45.7	0.103
DPB1*09:01	No	2393	98.3	24011	98.6	
	Yes	41	1.7	329	1.4	0.211
DPB1*100:01	No	2434	100	24339	100	
	Yes	0	0	1	0	1.000
DPB1*104:01	No	2424	99.6	24280	99.8	
	Yes	10	0.4	60	0.2	0.192
DPB1*135:01	No	2363	97.1	23669	97.2	
	Yes	71	2.9	671	2.8	0.693
DPB1*13:01	No	2276	93.5	22757	93.5	
	Yes	158	6.5	1583	6.5	1.000
DPB1*14:01	No	2361	97	23697	97.4	
	Yes	73	3	643	2.6	0.329
DPB1*17:01	No	2412	99.1	24099	99	
	Yes	22	0.9	241	1	0.761
DPB1*19:01	No	2406	98.8	24032	98.7	
	Yes	28	1.2	308	1.3	0.696
DPB1*21:01	No	2403	98.7	23924	98.3	
	Yes	31	1.3	416	1.7	0.130

DPB1*26:01	No	2434	100	24332	100	
	Yes	0	0	8	0	0.780
DPB1*296:01	No	2434	100	24332	100	
	Yes	0	0	8	0	0.780
DPB1*31:01	No	2429	99.8	24312	99.9	
	Yes	5	0.2	28	0.1	0.363
DPB1*414:01	No	2431	99.9	24332	100	
	Yes	3	0.1	8	0	0.116
DPB1*48:01	No	2433	100	24322	99.9	
	Yes	1	0	18	0.1	0.856

[a] Categorical variables were expressed as numbers (percent) and were analyzed using the Chi-square test.

Supplementary Table S6. The genotype frequencies of the *HLA-DQA1* alleles in the participants.

Variables		NSAID allergy n = 2434 (alleles)		Control n = 24340 (alleles)		*p* value[a]
		n	%	n	%	
DQA1*01:01	No	2390	98.2	23891	98.2	
	Yes	44	1.8	449	1.8	0.960
DQA1*01:02	No	2021	83	20294	83.4	
	Yes	413	17	4046	16.6	0.684
DQA1*01:03	No	2194	90.1	22058	90.6	
	Yes	240	9.9	2282	9.4	0.457
DQA1*01:04	No	2280	93.7	22711	93.3	
	Yes	154	6.3	1629	6.7	0.518
DQA1*01:05	No	2403	98.7	24081	98.9	
	Yes	31	1.3	259	1.1	0.396
DQA1*02:01	No	2394	98.4	23787	97.7	
	Yes	40	1.6	553	2.3	0.053
DQA1*03:01	No	2286	93.9	22517	92.5	
	Yes	148	6.1	1823	7.5	0.013
DQA1*03:02	No	2055	84.4	20606	84.7	
	Yes	379	15.6	3734	15.3	0.787
DQA1*03:03	No	2246	92.3	22276	91.5	
	Yes	188	7.7	2064	8.5	0.214
DQA1*04:01	No	2405	98.8	24050	98.8	
	Yes	29	1.2	290	1.2	1.000
DQA1*05:01	No	2254	92.6	22421	92.1	
	Yes	180	7.4	1919	7.9	0.415
DQA1*05:03	No	2420	99.4	24162	99.3	
	Yes	14	0.6	178	0.7	0.457
DQA1*05:05	No	2146	88.2	21571	88.6	
	Yes	288	11.8	2769	11.4	0.522
DQA1*05:06	No	2434	100	24338	100	
	Yes	0	0	2	0	1.000
DQA1*05:08	No	2408	98.9	24172	99.3	
	Yes	26	1.1	168	0.7	0.049
DQA1*06:01	No	2174	89.3	22165	91.1	
	Yes	260	10.7	2175	8.9	0.005

[a] Categorical variables were expressed as numbers (percent) and were analyzed using the Chi-square test.

Supplementary Table S7. The genotype frequencies of the *HLA-DQB1* alleles in the participants.

**Variables**		**NSAID allergy n = 2434 (alleles)**		**Control n = 24340 (alleles)**		***p* value[a]**
		**n**	**%**	**n**	**%**	
DQB1*02:01	No	2253	92.6	22421	92.1	
	Yes	181	7.4	1919	7.9	0.457
DQB1*02:02	No	2396	98.4	23850	98	
	Yes	38	1.6	490	2	0.146
DQB1*03:01	No	1839	75.6	18971	77.9	
	Yes	595	24.4	5369	22.1	0.008
DQB1*03:02	No	2268	93.2	22367	91.9	
	Yes	166	6.8	1973	8.1	0.028
DQB1*03:03	No	2052	84.3	20538	84.4	
	Yes	382	15.7	3802	15.6	0.947
DQB1*04:01	No	2274	93.4	22515	92.5	
	Yes	160	6.6	1825	7.5	0.105
DQB1*04:02	No	2404	98.8	24040	98.8	
	Yes	30	1.2	300	1.2	1.000
DQB1*05:01	No	2373	97.5	23747	97.6	
	Yes	61	2.5	593	2.4	0.886
DQB1*05:02	No	2167	89	21965	90.2	
	Yes	267	11	2375	9.8	0.061
DQB1*05:03	No	2340	96.1	23272	95.6	
	Yes	94	3.9	1068	4.4	0.245
DQB1*06:01	No	2133	87.6	21332	87.6	
	Yes	301	12.4	3008	12.4	1.000
DQB1*06:02	No	2350	96.5	23370	96	
	Yes	84	3.5	970	4	0.216
DQB1*06:03	No	2426	99.7	24263	99.7	
	Yes	8	0.3	77	0.3	1.000
DQB1*06:04	No	2433	100	24311	99.9	
	Yes	1	0	29	0.1	0.436
DQB1*06:09	No	2378	97.7	23853	98	
	Yes	56	2.3	487	2	0.355
DQB1*06:10	No	2424	99.6	24285	99.8	
	Yes	10	0.4	55	0.2	0.121

[a] Categorical variables were expressed as numbers (percent) and were analyzed using the Chi-square test.

Supplementary Table S8. The genotype frequencies of the *HLA-DRB1* alleles in the participants.

Variables		NSAID allergy n = 2434 (alleles)		Control n = 24340 (alleles)		*p* value[a]
		n	%	n	%	
DRB1*01:01	No	2423	99.5	24240	99.6	
	Yes	11	0.5	100	0.4	0.892
DRB1*01:02	No	2433	100	24338	100	
	Yes	1	0	2	0	0.648
DRB1*03:01	No	2254	92.6	22413	92.1	
	Yes	180	7.4	1927	7.9	0.383
DRB1*04:01	No	2425	99.6	24282	99.8	
	Yes	9	0.4	58	0.2	0.305
DRB1*04:03	No	2349	96.5	23320	95.8	
	Yes	85	3.5	1020	4.2	0.110
DRB1*04:04	No	2433	100	24331	100	
	Yes	1	0	9	0	1.000
DRB1*04:05	No	2258	92.8	22350	91.8	
	Yes	176	7.2	1990	8.2	0.112
DRB1*04:06	No	2372	97.5	23591	96.9	
	Yes	62	2.5	749	3.1	0.164
DRB1*04:10	No	2432	99.9	24329	100	
	Yes	2	0.1	11	0	0.759
DRB1*07:01	No	2394	98.4	23788	97.7	
	Yes	40	1.6	552	2.3	0.054
DRB1*08:02	No	2431	99.9	24324	99.9	
	Yes	3	0.1	16	0.1	0.537
DRB1*08:03	No	2210	90.8	22184	91.1	
	Yes	224	9.2	2156	8.9	0.594
DRB1*08:09	No	2408	98.9	24067	98.9	
	Yes	26	1.1	273	1.1	0.890
DRB1*09:01	No	2058	84.6	20632	84.8	
	Yes	376	15.4	3708	15.2	0.803
DRB1*10:01	No	2403	98.7	24080	98.9	
	Yes	31	1.3	260	1.1	0.407
DRB1*11:01	No	2205	90.6	22297	91.6	
	Yes	229	9.4	2043	8.4	0.094

DRB1*11:06	No	2433	100	24331	100	
	Yes	1	0	9	0	1.000
DRB1*12:01	No	2345	96.3	23375	96	
	Yes	89	3.7	965	4	0.490
DRB1*12:02	No	2161	88.8	22017	90.5	
	Yes	273	11.2	2323	9.5	0.009
DRB1*13:01	No	2426	99.7	24260	99.7	
	Yes	8	0.3	80	0.3	1.000
DRB1*13:02	No	2377	97.7	23824	97.9	
	Yes	57	2.3	516	2.1	0.517
DRB1*13:03	No	2433	100	24338	100	
	Yes	1	0	2	0	0.648
DRB1*13:12	No	2425	99.6	24232	99.6	
	Yes	9	0.4	108	0.4	0.714
DRB1*14:03	No	2432	99.9	24306	99.9	
	Yes	2	0.1	34	0.1	0.654
DRB1*14:04	No	2429	99.8	24291	99.8	
	Yes	5	0.2	49	0.2	1.000
DRB1*14:05	No	2377	97.7	23703	97.4	
	Yes	57	2.3	637	2.6	0.455
DRB1*14:54	No	2341	96.2	23378	96	
	Yes	93	3.8	962	4	0.792
DRB1*15:01	No	2242	92.1	22219	91.3	
	Yes	192	7.9	2121	8.7	0.179
DRB1*15:02	No	2392	98.3	23922	98.3	
	Yes	42	1.7	418	1.7	1.000
DRB1*15:04	No	2434	100	24337	100	
	Yes	0	0	3	0	1.000
DRB1*16:02	No	2285	93.9	23101	94.9	
	Yes	149	6.1	1239	5.1	0.032

[a] Categorical variables were expressed as numbers (percent) and were analyzed using the Chi-square test.
