# Supplementary figures and images for: Genetic Susceptibility of HLA Alleles to Non-Steroidal Anti-Inflammatory Drug Hypersensitivity in the Taiwanese Population
Source: Biomedicines. 2023 Dec 11;11(12):3273. doi: 10.3390/biomedicines11123273 (PMC10741656; doi:10.3390/biomedicines11123273)

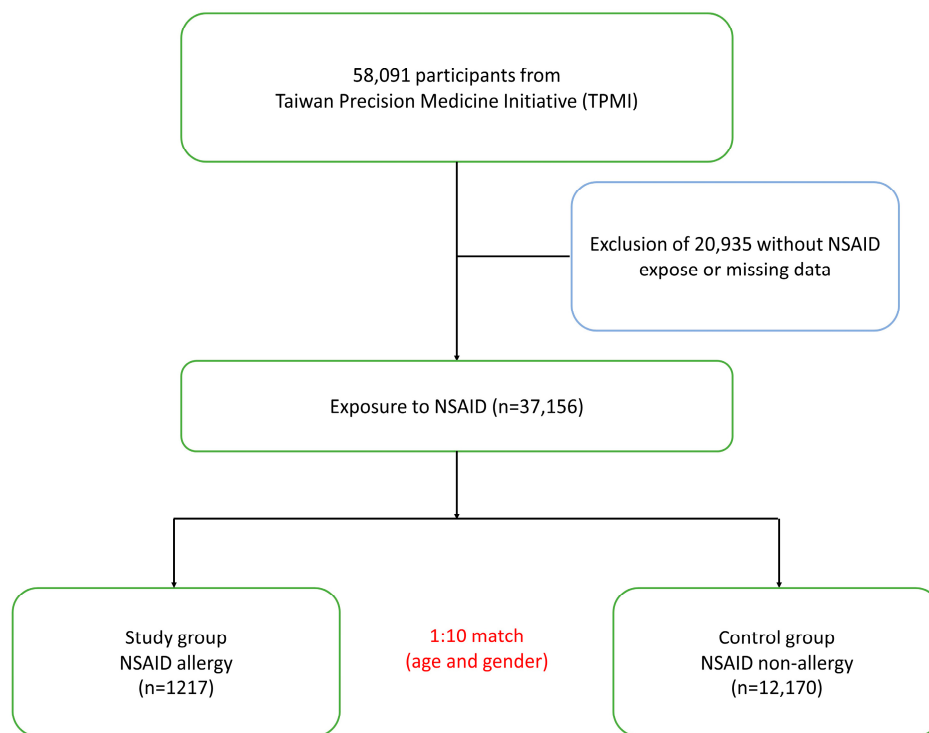

**Supplementary Figure S1.** Illustrative flow chart of the study design.

Supplement: Supplementary file 1 [file biomedicines-11-03273-s001.zip › Supplementary Figure S1.pdf]
